# Profiling of Phenolic Compounds and Antioxidant Activity of 12 Cruciferous Vegetables

**DOI:** 10.3390/molecules23051139

**Published:** 2018-05-10

**Authors:** Zhifeng Li, Hui Wen Lee, Xu Liang, Dong Liang, Qi Wang, Dejian Huang, Choon Nam Ong

**Affiliations:** 1 National Pharmaceutical Engineering Center for Solid Preparation in Chinese Herbal Medicine, Jiangxi University of Traditional Chinese Medicine, No. 818 Yunwan Road, Nanchang 330002, China; wangqilizhifeng@126.com (Z.L.); wangqi19760615@hotmail.com (Q.W.); 2 NUS Environmental Research Institute, National University of Singapore, 5A Engineering Drive 1, Singapore 117411, Singapore; erilhw@nus.edu.sg (H.W.L.); eriliaxu@nus.edu.sg (X.L.); 3 Saw Swee Hock School of Public Health, National University of Singapore, 12 Science Drive 2, Singapore 117549, Singapore; ephld@nus.edu.sg; 4 Food Science and Technology Program, Department of Chemistry, National University of Singapore, 3 Science Drive 3, Singapore 117543, Singapore; chmhdj@nus.edu.sg

**Keywords:** Brassicaceae vegetables, cruciferous vegetables, phenolic compounds, antioxidant activity, UHPLC-MS/MS, principal component analysis

## Abstract

The phenolic profiles of 12 cruciferous vegetables (pakchoi, choysum, Chinese cabbage, kailan, Brussels sprout, cabbage, cauliflower, broccoli, rocket salad, red cherry radish, daikon radish, and watercress) were studied with UHPLC-MS/MS. Antioxidant activity and total phenolic content (TPC) were also evaluated. A total of 74 phenolic compounds were identified, including 16 hydroxycinnamic acids and derivatives, and 58 flavonoids and derivatives. The main flavonoids identified were glycosylated quercetin, kaempferol and isorhamnetin, and the main hydroxycinnamic acids were ferulic, sinapic, caffeic and *p*-coumaric acids. Principal component analysis (PCA) revealed that the distribution of phenolic compounds in different genera of cruciferous vegetables was in accordance with their conventional taxonomy. The DPPH, ORAC and TPC values ranged from 1.11 to 9.54 µmoles Trolox equivalent/g FW, 5.34 to 32.92 µmoles Trolox equivalent/g FW, and 0.16 to 1.93 mg gallic acid equivalent/g FW respectively. Spearman’s correlation showed significant (*p* < 0.05) positive correlations between TPC, flavonoids and antioxidant activity.

## 1. Introduction

The Brassicaceae family consists of 350 genera and about 3500 species which include a wide range of horticultural crops that are of great economic significance, and constitute a major part of diets throughout the world [1]. The major nutritional constituents of Brassicaceae (cruciferous) vegetables are carbohydrates, proteins, vitamins like folic acid, ascorbic acid, provitamin A and tocopherols, and minerals including copper, iron, selenium, calcium, manganese and zinc [2]. In addition, they have negligible amounts of fat, which makes them an important constituent of a low-fat and heart-friendly diet. Besides macro- and micro-nutrients, cruciferous vegetables are also rich in bioactive, non-nutrient phytochemicals that have been linked to reducing the risk of several chronic diseases [3].

Cruciferous vegetables have received considerable attention in recent years due to their contribution to health improvements in the prevention of cancer, cardiovascular disease and other chronic diseases such as asthma, Alzheimer’s disease and metabolic disorders. Extensive epidemiological studies have shown inverse relationships between the consumption of cruciferous vegetables and the risk of various types of cancers including pancreatic, lung, colorectal, breast, ovarian and gastrointestinal cancer, and as such, cruciferous vegetables have become increasingly important in the area of cancer chemoprevention [2]. Recently, bioactive compounds of cruciferous vegetables have shown to play a role in the prevention of cardiovascular disease through the reduction of platelet aggregation, reduction of blood pressure, modulation of cholesterol synthesis and absorption and lipid profiles, and anti-inflammation [4].

Beside the characteristic glucosinolates and isothiocyanates, another major group of bioactive components present in cruciferous vegetables is the phenolic compounds [5], which refers to a large group of phytochemicals that comprise an aromatic ring bearing one or more hydroxyl substituents. Phenolic compounds are ubiquitous in the plant kingdom, and are strongly associated with the taste, color and species characteristics of vegetables. In addition, these plant secondary metabolites are good antioxidants, due to their hydrogen- or electron-donating abilities, as well as the capability to delocalize the unpaired electron within the aromatic structure [6]. Based on their structure, phenolic compounds can be categorized into different classes including simple phenols, phenolic acids, naphthoquinones, xanthones, stilbenes, flavonoids, lignans, and tannins. Among them, phenolic acids, flavonoids, and tannins are regarded as major dietary phenolic compounds [1,7].

Despite the perceived importance of phenolic compounds in maintaining good health, their comparative profile in cruciferous vegetables, especially those commonly consumed in Asia, is still lacking. Previous studies profiling phenolic compounds in cruciferous vegetables were either dedicated to one or more varieties of a vegetable subspecies, e.g., *Brassica rapa* [8,9], or several subspecies of a vegetable species, e.g., *Brassica* [10,11,12]. Moreover, studies involving the quantitative or semi-quantitative profiling of phenolic compounds in cruciferous vegetables are even scarcer.

Thus, the aim of this study was to profile the phenolic compounds—and measure the antioxidant activities—of 12 cruciferous vegetables commonly consumed in Asia—pakchoi (*Brassica. rapa* var. *chinensis*), choysum (*B. rapa* var. *parachinensis*), Chinese cabbage (*B. rapa* var. *pekinensis*), kailan (*B. oleracea* var. *alboglagra*), Brussels sprout (*B. oleracea* var. *gemmifera*), cabbage (*B. oleracea* var. *capitata*), cauliflower (*B. oleracea* var. *botrytis*), broccoli (*B. oleracea* var. *italica*), rocket salad (*Eruca sativa*), red cherry radish (*Raphanus sativus*), daikon radish (*Raphanus sativus*), and watercress (*Nasturtium officcinale*), so as to understand their distribution in the cruciferous vegetables.

## 2. Results and Discussion

### 2.1. Phenolic Compounds Identification

Profile of Brassicaceae vegetables, especially the *Brassica* species, have been well-studied. Phenolic compounds in vegetables exist in both free and conjugated forms, with the latter generally present in fresh vegetables [13]. The major classes of phenolic compounds found in cruciferous vegetables are flavonols—mainly *O*-glycosides of quercetin, kaempferol and isorhamnetin—and hydroxycinnamic acids—mainly ferulic, caffeic, *p*-coumaric and sinapic acids, found in conjugation with sugars or other hydroxycinnamic acids [1,14,15,16] and they are used for structural and chemical plant defense strategies [1,17].

Using UHPLC-Q-TOF-MS/MS, 74 chemical constituents were identified in 12 cruciferous vegetables, including 16 hydroxycinnamic acids and derivatives, and 58 flavonoids and derivatives. Of these compounds, the identification of 15 compounds were confirmed by comparing the retention time and MS spectra with their authentic standards, and the rest without available standard were tentatively identified by comparing their LC-Q-TOF-MS/MS data with previous studies [11,12]. The typical fragmentation behavior of *O*-glycoside flavonoids was observed in which the cleavage of the labile C–O bond resulted in the loss of the glycosyl moiety such as a glucose unit (162 Da) or a rutinose unit (308 Da) [18]. The loss of 162 Da is especially characteristic from flavonoid-3-*O*-(acyl)glycoside-7-*O*-hexoside, and has been widely described in different *Brassica* species [8]. In addition, the loss of one caffeic acid moiety (162 Da) in chlorogenic acid derivatives due to ester bond cleavage, and the loss of a dihexoxyl group (324 Da) were also observed [15].

### 2.2. Method Validation

**Figure 1:**
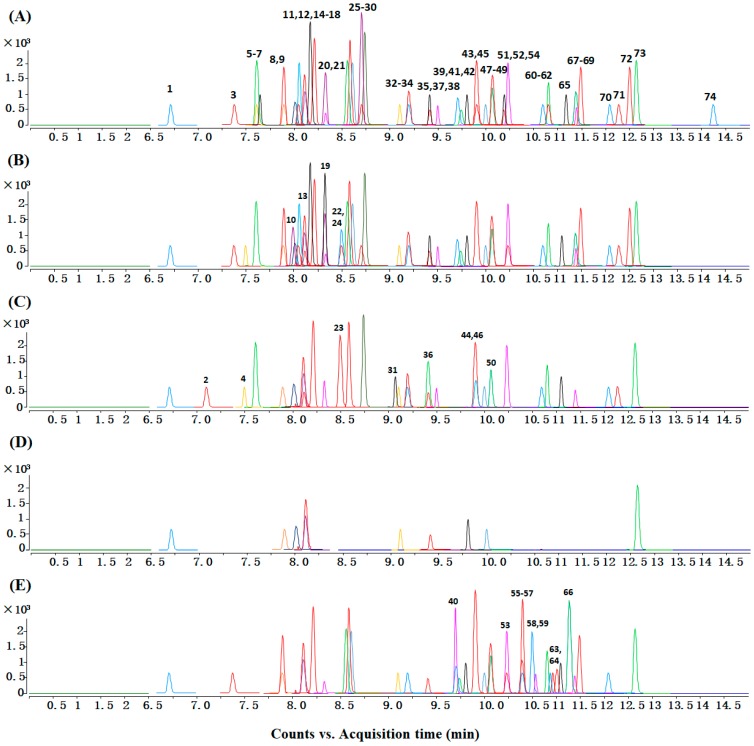
MRM chromatograms of phenolic compounds in (**A**) *Brassica rapa*; (**B**) *B. oleracea*; (**C**) *Eruca*; (**D**) *Raphanus*; and (**E**) *Nasturtium* species. The identity of the peaks are listed in Table S1.

Quantitation of phenolic compounds was done in the MRM mode using UHPLC-QqQ-MS/MS (Supplementary Table S1). For phenolic compounds without available standards, cynaroside was used to semi-quantify kaempferol glycosides, quercetin-3-*O*-glucoside was used to semi-quantify quercetin glycosides, isorhamnetin-3-*O*-rutinoside was used to semi-quantify isorhamnetin glycosides, sinapic acid was used to semi-quantify sinapic acid derivatives, and ferulic acid was used to semi-quantify ferulic acid derivatives. In addition, the MRM chromatograms of the 12 cruciferous vegetables, according to their species/subspecies, are shown in Figure 1.

The linearity, limit of detection (LOD), reproducibility and recoveries for the quantification method using UHPLC-QqQ-MS/MS are shown in Supplementary Table S2. Linear calibration curves of compounds with available standards were obtained by plotting the ratio of the peak area of analyses to the peak area of the internal standard against the corresponding concentration. The equation and the coefficient of determination (*R*^2^) of the calibration curves were determined using a linear regression model. Good linear correlations were obtained at the present chromatographic conditions for the standards, with the *R*^2^ values all above 0.9953. The LODs, measured with a signal-to-noise ratio (S/N) of 3, ranged from 0.01 to 0.24 ng/mL, indicating that the analytical method was sensitive enough for the quantitative determination of the compounds in cruciferous vegetables. The relative standard deviation (RSD) values for reproducibility were in the range of 0.89% to 6.48%, and the recovery rates for low and high spiked concentrations were from 71.63% to 111.31%, and 91.53% to 114.78% respectively. In all, the results indicated that the analytical method demonstrated good sensitivity, reproducibility, and recovery.

### 2.3. Phenolic Compounds Profiling of 12 Cruciferous Vegetables

The validated analytical method was used to obtain the phenolic compound profile of 12 cruciferous vegetables. The concentrations of the phenolic compound in the vegetables were determined from the peak area obtained in the MRM mode by interpolation from the respective standard calibration curve, and expressed as micrograms per gram dry weight of vegetable (Table 1).

The chemical constituents in the 12 cruciferous vegetables were broadly categorized into two different groups—hydroxycinnamic acids and derivatives, and flavonoids and derivatives. Hydroxycinnamic acids and derivatives included compounds with two or more hydroxycinnamic acids residues which may also be glycosylated. The flavonoids and derivatives group consisted of aglycone flavonoids, flavonoid glycosides and flavonoids. Ferulic acid, sinapic acid, caffeic acid, and *p*-coumaric acid were found in all cruciferous vegetables except daikon radish and red cherry radish, which did not contain sinapic acid. In addition, iso-sinapic acid was only detected in Brussels sprout, cabbage, Chinese cabbage, red cherry radish and watercress. In all, cauliflower contained the highest amount of phenolic acid (5.70 mg/g dry weight), while daikon radish contained the least (0.47 mg/g dry weight).

A total of 16 hydroxycinnamic acid derivatives were detected in the 12 vegetables; for 9 of these, standards were available. However, as 4- and 5-feruloylquinic acids co-eluted, it was impossible to discriminate the two compounds. As such, further quantification of the peak was done using only 4-feruloylquinic acid. Cabbage contained the highest content of hydroxycinnamic acids and derivatives (46.02 mg/g dry weight), while daikon radish (1.02 μg/g dry weight) and red cherry radish (1.68 μg/g dry weight) contained the lowest. In daikon radish and red cherry radish, the predominant hydroxycinnamic acid derivatives were feruloylquinic and caffeoylquinic acid derivatives, while the predominant ones in the rest of the vegetables were ferulic and sinapic acid derivatives conjugated with gentiobiose. Among feruloylquinic and caffeoylquinic acid derivatives, the content of 5-caffeoylquinic acid and 3-feruloylquinic acid in the vegetables were the highest. Among the ferulic and sinapic acid derivatives conjugated with gentiobiose, the contents 1,2-disinapoylgentiobiose and 1-sinapoyl-2-ferulicgentiobiose were higher than those of other compounds. Interestingly, 3-feruloylquinic acid, 1,2′-disinapoyl-2-feruloylgentiobiose, and 1-sinapoyl-2-2′-diferuloylgentiobiose were not detected in watercress, and 1,2-diferuloylgentiobiose and 1-sinapoyl-2-2′-diferuloylgentiobiose were not detected in rocket salad, suggesting that these phenolic compounds could be used as biomarkers to distinguish *Nasturtium* and *Eruca* from other genera. In addition, the hydroxycinnamic acid derivatives content of vegetables in *B. oleracea* subspecies (broccoli, Brussels sprout, cabbage, cauliflower and kailan) were found to be generally higher than those of other genera and *B. rapa* subspecies.

Flavonoids are present in the epidermis of leaves and fruits and have a wide range of important roles as secondary metabolites, such as absorption of UV radiation and strong light, protection against insect predation and microbes, attraction of insect pollinators, and inhibition of reactive oxygen species generation through antioxidative actions [19,20]. Among the 12 cruciferous vegetables, the main flavonoids (flavonols) were found to be mainly *O*-glycosides of quercetin, kaempferol and isorhamnetin. As there could be many isomers of flavonoid glycosides due to glycosylation at different positions, without the respective standards, flavonoid glycoside isomers could only be distinguished from them by their retention time, and could only be semi-quantified using cynaroside for kaempferol glycosides, quercetin-3-*O*-glucoside for quercetin glycosides and isorhamnetin-3-*O*-rutinoside for isorhamnetin.

Among the vegetables studied, it is interesting to observe that each has its unique phenolic profile. While quercetin aglycone was present in all vegetables, isorhamnetin aglycone was only found in choysum (1.61 μg/g dry weight) and pakchoi (0.44 μg/g dry weight) at very low levels, and kaempferol aglycone was absent in all 12 vegetables. However, kaempferol glycosides were in greater abundance than quercetin and isorhamnetin glycosides. Daikon radish and red cherry radish did not contain quercetin, kaempferol or isorhamnetin glycosides; Brussels sprouts, cabbage, cauliflower, and kailan did not contain isorhamnetin glycosides, and cabbage did not contain much quercetin glycosides (0.07 μg/g dry weight of quercetin-diglucoside). In previous work, the sugar moiety in *Brassica* vegetables was found to be glucose, occurring as mono-, di-, tri-, tetra-, and penta-glucosides [15], and this was also the case for the 12 cruciferous vegetables analyzed. Interestingly, rutin (quercetin-3-*O*-rutinoside) and nicotiflorin (kaempferol-3-*O*-rutinoside) were also identified in the vegetables, but they were only found mainly in watercress, while the former was found to be present in rocket salad at a very low level.

The *O*-glycosides of quercetin, kaempferol and isorhamnetin were also found to be acylated with hydroxycinnamic acids, such as ferulic, sinapic, *p*-coumaric and caffeic acids. Most of the identified flavonoids acylated with hydroxycinnamic acid were kaempferol glycosides, however some quercetin glycosides acylated with ferulic, caffeic, and sinapic acids were also identified. Similar to the trend obtained for flavonoids, daikon radish and red cherry radish did not contain any flavonoids acylated with hydroxycinnamic acid. In essence, daikon radish and red cherry radish did not contain any flavonoids except quercetin. In all, pakchoi contained the highest amount of flavonoids (1.40 mg/g dry weight), while cabbage contained the lowest (13.44 μg/g dry weight). Overall, daikon radish exhibited the lowest concentration of total phenolic compounds (484.73 µg/g dry weight), while cauliflower showed the highest (47.84 mg/g dry weight).

### 2.4. PCA Analysis

Chemotaxonomy has garnered attention as a modern approach to plant classifications based on their chemical constituents, due to its relative ease of working methodology (Singh, 2016). In particular, phenolic compounds in plants could be useful for chemotaxonomic classification, as such secondary metabolites are restricted and specific to taxonomically related species. By identifying the major composition and structure of chemical constituents in plants, characteristic compounds within the plant species and genus can be known, thus enabling the evaluation of differences in chemotaxonomic features between various plant species and genera. Previous works have reported the use of glucosinolates content as a chemotaxonomy marker in Brassicaceae vegetables, mainly *Brassica* species [21,22], but works reporting the use of phenolic compounds as a chemical biomarker for Brassicaceae vegetables are few.

**Figure 2:**
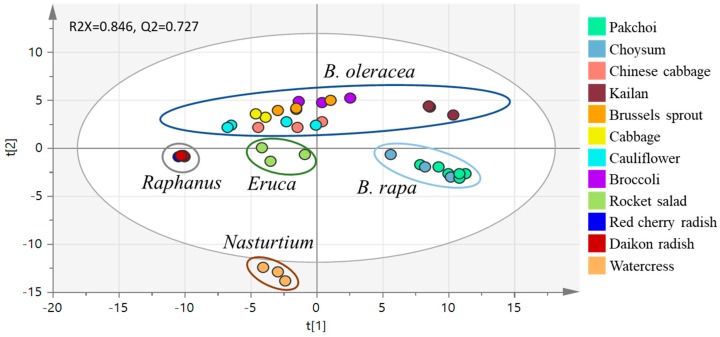
PCA analysis results obtained from the phenolic constituents of the three batches of 12 cruciferous vegetables displaying principle components 1 and 2. Contribution to overall variation were PC1 40.6% and PC2 17.6%.

In this study, PCA was used to characterize the broad patterns of changes in concentrations of 74 chemical constituents, to allow easy visualization of the complex data according to the similarity of grouped data. PCA modeling (Figure 2) using the data set of 74 compounds revealed a clear separation of the vegetables into five groups, according to the species of the vegetables except for kailan and Chinese cabbage, which were relatively closer to the *B. rapa* and *B. oleracea* subspecies respectively. Nonetheless, the PCA plot was generally able to discriminate between vegetables at the genus level, suggesting the phenolic compound profiles could be used as a potential biomarker for the classification of cruciferous vegetables.

Supervised multivariate OPLS-DA was applied to achieve maximum separation among different groups. The features with VIP > 1.2 and *p* < 0.05 were selected from each comparison and combined for identification of differential components. Finally, eleven differential components were obtained, including five hydroxycinnamic acids and derivatives (sinapic acid, 1,2,2′-trisinapoylgentiobiose, 1,2′-disinapoyl-2-feruloylgentiobiose, 1-sinapoyl-2-feruloylgentiobiose, 1,2-disinapoylgentiobiose), and six flavonoids and derivatives (quercetin-diglucoside, quercetin-triglucoside, quercetin-3-*O*-glucoside, isorhamnetin-diglucoside, kaempferol-3-*O*-caffeoyldiglucosi de-7-*O*-diglucoside, rutin). Therefore, these constituents were chosen as the biomarkers to distinguish the differences in phenolic compounds profiles of the cruciferous vegetables in the five genera. Though the PCA plot was sufficient to show that the phenolic compounds profile of vegetables in different genera were varied, subsequent analyses of more varieties of vegetables within the same genera would greatly enhance confidence in observations of biomarker differences amongst them.

### 2.5. DPPH, ORAC and TPC Assays and Its Relations to Phenolic Compounds

Cruciferous vegetables are a rich source of dietary antioxidants, including water-soluble and water-insoluble antioxidants [5]. In this study, the hydrophilic antioxidant activity, measured by DPPH and ORAC, and TPC of the 12 cruciferous vegetables were studied; the results are shown in Table 2. The range for the DPPH radical scavenging activity, ORAC and TPC assays in the 12 cruciferous vegetables varied from 1.11 to 9.54 μmol TE/g FW, 3.45 to 32.92 μmol TE/g FW, and 0.16 to 1.93 mg GAE/g FW respectively.

Despite differences between ORAC and DPPH results, the trend was clear among the 12 vegetables. In both methods, rocket salad, watercress and Brussels sprouts possessed the highest antioxidant activity, followed by kailan, while cabbage, Chinese cabbage and daikon radish possessed the lowest. In addition, there did not appear to be a species-specific trend in terms of antioxidant activity and total phenolic content among the cruciferous vegetables. Our results were in agreement with previous studies, for example, Kaur and Kapoor [23], who by examining 34 Asian vegetables showed that Brussels sprouts had high antioxidant activity, followed by cabbage (with medium antioxidant activity), and cauliflower and daikon radish (with low antioxidant activities).

**Figure 3:**
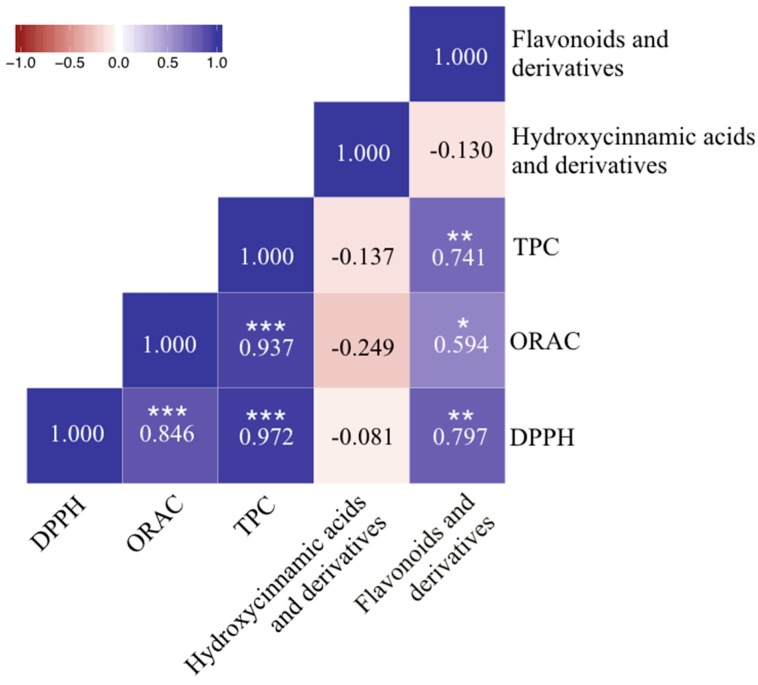
Spearman’s correlation coefficients (two-tailed) for the relationships between antioxidant capacity (DPPH and ORAC), TPC, hydroxycinnamic acids and derivatives, and flavonoids and derivatives in cruciferous vegetables. * Correlation is significant at 0.01 ≤ *p* < 0.05; ** Correlation is significant at 0.001 ≤ *p* < 0.01; *** Correlation is significant at *p* < 0.001.

To observe the relationship between antioxidant activity and the chemical composition of the vegetables, correlation analyses were performed for DPPH, ORAC, and TPC, and the two major groups of chemical constituents as well as the total amount of phenolic compound were evaluated in this study (coined total phenolic compounds, representing the sum of hydroxycinnamic acids and derivatives, and flavonoids and derivatives) (Figure 3). High and significant correlations between TPC and antioxidant activity were evaluated using DPPH and ORAC as Spearman’s coefficient correlation, *ρ*, were determined to be 0.972 and 0.937 respectively, suggesting that TPC is a good predictor of in vitro antioxidant activity. However, the TPC assay using the Folin-Ciocalteu reagent is an indirect measurement of the total phenolic content, as it measures the total reducing capacity of a sample and is prone to interferences from non-phenolic reducing agents such as ascorbic acid, citric acid, simple sugars and amino acids [24]. In addition, the correlation between DPPH and TPC was stronger than that between TPC and ORAC or between DPPH and ORAC (*ρ* = 0.846), due to the different reaction mechanisms of both assays. While ORAC measures the ability of the antioxidant to donate hydrogen atoms, DPPH and TPC are based on the electron donation ability of the antioxidant [25].

Comparing the two groups of chemical constituents determined by UHPLC-QqQ-MS/MS, flavonoids and derivatives showed significant positive correlations to DPPH, ORAC, and TPC assays with *ρ* values of 0.797, 0.594 and 0.741 respectively, further implying that they are major contributors to the antioxidant properties of vegetables, while no correlation was found between hydroxycinnamic acids and derivatives contents and antioxidant activity.

## 3. Materials and Methods

### 3.1. Chemicals and Reagents

Commercial standards including *p*-coumaric acid, ferulic acid, caffeic acid, sinapic acid, 3-caffeoylquinic acid, 4-caffeoylquinic acid, 5-caffeoylquinic acid, 3-feruloylquinic acid, 4-feruloylquinic acid, 5-feruloylquinic acid, kaempferol, isorhamnetin, myricetin, apigenin, quercetin, quercetins (quercetin-3-*O*-rhamnoside), isoquercitrin (quercetin-3-*O*-glucoside), rutin (quercetin-3-*O*-rutinoside), cynaroside (luteolin-7-*O*-glucoside), narcissin (isorhamnetin-3-*O*-rutinoside) and nicotiflorin (kaempferol-3-*O*-rutinoside) were purchased from Chengdu Push Bio-technology Co., Ltd. (Chengdu, China). Folin-Ciocalteu’s phenol reagent, sodium carbonate, gallic acid, 2,2-diphenyl-1-picrylhydrazyl (DPPH), potassium dihydrogen phosphate, and dipotassium hydrogen phosphate were obtained from Sigma (St. Louis, MO, USA). Trolox, fluorescein and 2,2′-azobis(2-methylpropionamidine) dihydrochloride (AAPH) were purchased from Acros Organics (Morris Plains, NJ, USA), Fluka Analytical (Morris Plains, NJ, USA) and Manchester Organics Limited (Cheshire, UK) respectively. Acetonitrile and methanol of LC-MS grade were purchased from Fisher Scientific (Pittsburgh, PA, USA). LC-MS grade formic acid was purchased from Sigma (St. Louis, MO, USA). Acetone, methanol and glacial acetic acid of analytical grade were purchased from Sigma (St. Louis, MO, USA). Ultrapure water (18.2 MΩ/cm) was produced by Siemens Ultra Clear TWF water purification system (Munich, Germany).

### 3.2. Sample Collection and Preparation

Batches of pakchoi, choysum, Chinese cabbage, kailan, Brussels sprout, cabbage, cauliflower, broccoli, rocket salad, red cherry radish, daikon radish, and watercress were purchased from various supermarkets in Singapore on different days (*n* = 3). The vegetables were washed with tap water and cut into smaller pieces before being freeze-dried using Labconco FreeZone freeze dryer (Kansas City, MO, USA). The freeze-dried samples were blended under dim light, and stored in light-protected centrifuge tubes at −80 °C before analysis.

### 3.3. Standards Preparation

Stock solutions of individual standards (1 mg/mL) were prepared by dissolving the compounds in methanol or 50% methanol (*v*/*v*). A mixed standard solution containing 50 μg/mL of individual standards was prepared by dilution of the stock solutions with methanol. The mixed standard was further diluted with methanol to obtain a series of working standard solutions for the construction of calibration curves. Stock solution of apigenin (internal standard) was prepared at 1 mg/mL in methanol. The final working standard solutions contained 50 ng/mL of internal standard. All solutions were stored at −20 °C.

### 3.4. Phenolic Compounds Extraction

Vegetable powder (0.5 g) was extracted twice with 15 mL of 70% (*v*/*v*) methanol, with the internal standard added into the extraction solvent during the first extraction. After the addition of the solvent, the mixture was vortexed for 30 s, followed by sonication for 20 min at room temperature. After sonication, the mixture was centrifuged at 20,000× *g* for 5 min to collect the supernatant. The pooled supernatant was filtered through a 0.22 μm PTFE membrane before chromatographic analysis.

### 3.5. Phenolic Compounds Identification and Quantification

Preliminary identification of phenolic compounds was carried out using an Agilent Technologies 1290 Infinity II LC system equipped with a 6540 UHD Accurate-Mass Q-TOF LC/MS (Santa Clara, CA, USA) with a dual AJS ESI interface. Subsequently, quantification of phenolic compounds was performed using an Agilent Technologies 1290 Infinity II LC system (Santa Clara, CA, USA) coupled to an Agilent 6490 triple quadrupole (QqQ) mass spectrometer with a Jet Stream ESI ion source (G1958-65138). Separation of phenolic compounds was achieved on a Zorbax RRHD XDB-C18 column (100 mm × 2.1 mm, 1.8 μm particle size) from Agilent (Santa Clara, CA, USA). The auto-sampler and column were maintained at 4 °C and 35 °C respectively, with an injection volume of 5 μL. The mobile phases used were 0.1% formic acid in water (solvent A) and 0.1% formic acid in acetonitrile (solvent B) at a flow of 0.4 mL/min, with the following gradient elution program: 2% B (0–4 min), 2–80% B (4–25 min), 80–95% B (25–36 min), and reconditioned with 2% B (36–40 min). Electrospray ionization was performed in negative ion mode with the following source parameters: drying gas (N_2_) temperature of 290 °C with a flow of 11 L/min, nebulizer gas pressure of 40 psi, sheath gas temperature of 350 °C with a flow of 12 L/min and capillary voltage of 3000 V. Mass spectra were acquired in the multiple reaction monitoring (MRM) mode. Agilent MassHunter software version B.05.00 (Santa Clara, CA, USA) was used for data acquisition and processing.

### 3.6. Method Validation for Phenolic Compounds Chromatographic Analysis

The analytical method for the quantification of phenolic compounds in the vegetables was validated for linearity, limit of detection (LOD), reproducibility and recovery. Briefly, standards were dissolved individually in methanol and diluted to provide a series of standard solutions with gradient concentration to obtain the calibration curves. Method reproducibility and recovery were carried out using mixed vegetable powder containing all 12 cruciferous vegetables. To validate method reproducibility, six independent phenolic compounds extracts were analyzed. The recoveries of the standards in the mixed vegetable powder were determined by spiking three defined amounts (approximately equivalent to 0.8, 1.0 and 1.2 times of the concentration of the matrix) into the mixed vegetable powder, in triplicate, for extraction and analysis, as described earlier.

### 3.7. Sample Preparation for DPPH, ORAC and Total Phenolic Content Assays

Vegetable samples extraction were carried out according to previous publication [26] with some modifications. Vegetable powder (31.25–62.5 mg) was extracted thrice with 500 μL acetone/water/acetic acid (AWA; 70:29.5:0.5, *v*/*v*), and sonicated for 15 min (Elma S60H Ultrasonicator, Elma Schmidbauer GmbH, Singen, Germany). After sonication, the mixture was centrifuged at 20,000× *g* for 5 min to collect the supernatant. Each batch of sample was extracted and assayed in duplicate. The pooled supernatant was used for DPPH, ORAC, and total phenolic content assays.

### 3.8. DPPH Radical Scavenging Activity

The DPPH radical-scavenging activity was determined using the microplate method described by Bobo-García, et al. [27] with minor modifications. Diluted vegetable extract (20 μL) was added to 180 μL of 0.2 mM DPPH solution (from 1.0 mM stock) in methanol on a polystyrene 96-well microplate (Corning, New York, NY, USA). After 2 h in the dark at room temperature, the microplate was shaken for 5 s and the absorbance was measured at 515 nm on BioTek SynergyMx microplate reader (Winooski, VT, USA). A calibration curve of %DPPH quenched against concentration was set up using Trolox as a standard at 50 to 500 μM (from 0.02 M stock). The %DPPH quench was calculated from Equation (1), where *A*_sample_ is the absorbance of the extract or Trolox with DPPH after 2 h, *A*_blank_ is the absorbance of 200 μL methanol after 2 h, and *A*_control_ is the absorbance of AWA with DPPH after 2 h. Final results were expressed as micromole Trolox equivalents per gram fresh weight of vegetables (μmol TE/g FW).

(1) $$\%\;\mathrm{DPPH}\;\mathrm{quenched}=\left[1-\left(\frac{A_{\mathrm{sample}}-A_{\mathrm{blank}}}{A_{\mathrm{control}}-A_{\mathrm{blank}}}\right)\right]\times 100$$

### 3.9. Oxygen Radical Absorbance Capacity (ORAC) Assay

Oxygen radical absorbance capacity (ORAC) assay was carried out on a BioTek Synergy HT microplate reader (Winooski, VT, USA) according to procedures previously described [28]. Data were expressed as micromole Trolox equivalents per gram fresh weight of vegetable (μmol TE/g FW).

### 3.10. Total Phenolic Content (TPC) Assay

Total phenolic content (TPC) of the vegetable extracts was determined using Folin-Ciocalteau reagent by a microplate method described previously [29]. Gallic acid was used as a standard at 0.016 to 0.25 g/L (from 1.0 g/L stock). The absorbance was measured at 765 nm on BioTek SynergyMx microplate reader (Winooski, VT, USA) after 2 h in the dark at room temperature. Data were expressed as milligram gallic acid equivalents per gram fresh weight of vegetable (mg GAE/g FW).

### 3.11. Statistical Analyses

Statistical analyses and Spearman’s correlation were performed using IBM SPSS Statistics at a significance level of 0.05 (two-tailed). A principal component analysis (PCA) was performed using SIMCA 14.0 (Sartorius Stedim Data Analytics AB, UMEÅ, Sweden), in which chemical constituents with VIP values greater than 1.2 and significant *p*-values (<0.05) were selected as discriminating compounds among the five groups of samples.

## 4. Conclusions

In conclusion, 74 phenolic compounds, including 16 hydroxycinnamic acids and derivatives, and 58 flavonoids and derivatives, were detected and quantified in 12 cruciferous vegetables using UHPLC-Q-TOF-MS/MS and UHPLC-QqQ-MS/MS. The main flavonoids identified were glycosylated quercetin, kaempferol and isorhamnetin, and the main hydroxycinnamic acids were ferulic, sinapic, caffeic and *p*-coumaric acids. Using PCA analysis, the profile of phenolic compounds from the 12 vegetables can be grouped and were observed to follow the traditional taxonomic classification, hence highlighting the potential use of phenolic compound profiles for the taxonomical classification of cruciferous plants. Antioxidant activity and total phenolic content were the highest in rocket salad, watercress and Brussels sprouts. In addition, total phenolic content, as well as flavonoids content, may be good predictors of the antioxidant activity of vegetables, as both of them are positively correlated with antioxidant activity in vegetables.

## Figures and Tables

**Table 1:** Concentration of 74 phenolic compound in 12 cruciferous vegetables. Results were expressed as mean ± standard error of mean (SEM) in μg/g DW. (*n* = 3, ND: not detected).

Class	Constituent	Pakchoi	Choysum	Chinese Cabbage	Kailan	Brussels Sprout	Cabbage	Cauliflower	Broccoli	Rocket Salad	Red Cherry Radish	Daikon Radish	Water-Cress
Hydroxy- cinnamic acids and derivatives	Ferulic acid	1.81 ± 0.21	2.37 ± 0.64	4.58 ± 1.55	4.68 ± 1.68	0.72 ± 0.33	1.43 ± 0.70	1.46 ± 0.72	1.95 ± 0.46	0.89 ± 0.09	0.28 ± 0.02	0.26 ± 0.07	7.24 ± 3.08
	Sinapic acid	2.94 ± 0.63	5.78 ± 0.51	5.69 ± 2.44	8.06 ± 4.21	4.69 ± 1.73	9.20 ± 2.25	15.16 ± 5.68	6.66 ± 1.28	45.44 ± 16.22	ND	ND	1.90 ± 0.43
	Iso-sinapic acid	ND	ND	1.93 ± 0.59	ND	0.49 ± 0.50	1.50 ± 0.83	ND	ND	ND	0.40 ± 0.03	ND	0.33 ± 0.26
	Caffeic acid	1.56 ± 0.28	2.58 ± 0.02	0.69 ± 0.37	1.79 ± 0.29	2.13 ± 0.85	2.00 ± 0.74	0.73 ± 0.40	0.59 ± 0.04	0.79 ± 0.53	0.54 ± 0.23	0.08 ± 0.09	1.78 ± 1.00
	*p*-Coumaric acid	0.15 ± 0.06	2.33 ± 1.24	0.34 ± 0.36	0.25 ± 0.18	0.69 ± 0.61	0.73 ± 0.51	0.59 ± 0.31	1.38 ± 0.50	0.05 ± 0.11	0.01 ± 0.04	0.19 ± 0.30	14.17 ± 5.20
	1,2-Diferuloyl gentiobiose	0.63 ± 0.11	2.42 ± 1.52	2.16 ± 0.44	118.55 ± 9.69	13.45 ± 11.25	2.65 ± 0.67	1.70 ± 1.56	125.75 ± 39.35	ND	ND	ND	2.11 ± 1.28
	1-Sinapoyl-2-2′diferuloyl gentiobiose	0.14 ± 0.02	0.20 ± 0.08	0.30 ± 0.10	11.33 ± 0.79	1.99 ± 1.68	1.13 ± 0.37	0.34 ± 0.27	7.31 ± 2.17	ND	ND	ND	ND
	1,2,2′-Trisinapoyl gentiobiose	58.61 ± 8.88	157.71 ± 16.19	138.72 ± 33.18	413.88 ± 21.06	1101.56 ± 157.39	876.00 ± 166.76	237.21 ± 48.15	648.78 ± 46.04	263.33 ± 70.93	ND	ND	0.06 ± 0.09
	1,2′-Disinapoyl-2-feruloyl gentiobiose	19.24 ± 5.04	27.67 ± 6.05	34.34 ± 9.23	509.36 ± 31.26	312.67 ± 149.90	184.51 ± 37.16	30.56 ± 16.66	305.62 ± 50.75	0.69 ± 0.16	ND	ND	ND
	1,2-Disinapoyl gentiobiose	1741.74 ± 417.34	3318.31 ± 383.26	7617.68 ± 3501.44	8610.26 ± 1248.64	29,214.88 ± 1147.33	40,030.20 ± 16,038.61	5847.86 ± 1884.12	17,839.37 ± 5576.89	8919.02 ± 3174.88	ND	ND	6315.94 ± 3250.87
	1-Sinapoyl-2-feruloyl gentiobiose	546.28 ± 113.26	615.91 ± 106.82	1548.59 ± 894.68	11,811.37 ± 1230.59	7266.49 ± 2408.39	4786.59 ± 1807.72	844.80 ± 604.89	15,724.93 ± 5151.71	21.18 ± 5.59	ND	ND	433.82 ± 115.63
	4 or 5-Feruloyl quinic acid	0.65 ± 0.20	2.21 ± 0.18	5.06 ± 2.62	13.18 ± 1.08	1.50 ± 0.57	0.84 ± 0.43	0.11 ± 0.03	1.22 ± 0.89	0.03 ± 0.01	0.03 ± 0.01	0.04 ± 0.00	0.03 ± 0.00
	3-Caffeoyl quinic acid	4.08 ± 1.31	14.30 ± 4.11	23.60 ± 18.16	64.04 ± 4.61	9.01 ± 5.20	2.61 ± 2.22	2.87 ± 2.81	42.36 ± 10.52	1.15 ± 1.09	0.02 ± 0.01	0.02 ± 0.01	0.12 ± 0.02
	4-Caffeoyl quinic acid	9.27 ± 2.87	29.43 ± 3.47	30.86 ± 7.62	150.42 ± 13.57	114.65 ± 56.30	25.15 ± 6.19	5.43 ± 1.30	23.56 ± 1.03	0.19 ± 0.06	008 ± 0.01	0.10 ± 0.00	0.07 ± 0.01
	3-Feruloyl quinic acid	7.22 ± 1.85	21.87 ± 3.48	44.47 ± 9.71	128.84 ± 6.66	24.40 ± 0.62	3.61 ± 2.66	1.12 ± 0.29	11.16 ± 3.77	0.46 ± 0.06	0.16 ± 0.02	0.16 ± 0.01	ND
	5-Caffeoyl quinic acid	77.68 ± 34.49	174.53 ± 39.31	149.08 ± 72.37	745.86 ± 77.58	418.52 ± 157.09	93.46 ± 30.74	100.98 ± 58.86	206.43 ± 66.65	0.39 ± 0.16	0.16 ± 0.01	0.17 ± 0.01	0.16 ± 0.01
Total hydroxycinnamic acids and derivatives		2472.00 ± 433.94	4377.62 ± 400.24	9608.09 ± 3614.89	22,591.87 ± 1755.35	38,487.84 ± 2681.78	46,021.61 ± 16,141.09	7090.92 ± 1980.38	34,947.07 ± 7592.93	9253.61 ± 3175.72	1.68 ± 0.24	1.02 ± 0.32	6777.73 ± 3252.94
Flavonoids and derivatives	Kaempferol-triglucoside	224.70 ± 13.88	216.77 ± 26.66	5.06 ± 0.43	94.11 ± 18.46	16.98 ± 4.57	6.12 ± 3.42	1.07 ± 0.88	3.42 ± 1.88	0.80 ± 0.62	ND	ND	38.68 ± 10.81
	Kaempferol-diglucoside	9.19 ± 1.11	6.34 ± 3.69	0.31 ± 0.03	2.94 ± 0.90	ND	ND	ND	0.46 ± 0.24	ND	ND	ND	0.10 ± 0.09
	Kaempferol-triglucoside	10.04 ± 3.13	9.72 ± 2.60	ND	4.56 ± 1.86	ND	ND	ND	ND	ND	ND	ND	ND
	Kaempferol-glucoside	5.38 ± 1.22	4.33 ± 1.37	ND	2.17 ± 1.10	ND	ND	ND	1.27 ± 0.43	1.81 ± 1.06	ND	ND	0.79 ± 0.65
	Kaempferol-glucoside	3.53 ± 1.35	2.51 ± 1.01	ND	17.94 ± 1.82	0.84 ± 0.76	1.35 ± 0.61	0.41 ± 0.47	5.89 ± 2.60	9.13 ± 1.88	ND	ND	ND
	Kaempferol-diglucoside	2.29 ± 0.30	3.01 ± 0.21	0.19 ± 0.44	16.02 ± 1.37	ND	ND	0.87 ± 0.88	4.46 ± 1.20	57.27 ± 2.96	ND	ND	ND
	Kaempferol-diglucoside	12.55 ± 2.30	15.61 ± 0.68	0.84 ± 0.74	12.58 ± 1.45	0.44 ± 0.58	0.01 ± 0.21	0.38 ± 0.57	ND	ND	ND	ND	32.89 ± 4.90
	Quercetin-3-*O*-glucoside	2.36 ± 0.28	1.97 ± 0.71	0.27 ± 0.14	0.64 ± 0.24	ND	ND	ND	0.38 ± 0.40	24.79 ± 12.36	ND	ND	42.83 ± 7.13
	Quercetin-triglucoside	0.56 ± 0.31	0.36 ± 0.20	ND	0.26 ± 0.08	ND	ND	ND	ND	ND	ND	ND	ND
	Quercetin-triglucoside	ND	ND	ND	ND	ND	ND	ND	ND	0.39 ± 0.05	ND	ND	ND
	Quercetin-triglucoside	1.22 ± 0.34	1.11 ± 0.43	ND	0.90 ± 0.28	0.05 ± 0.03	ND	ND	ND	ND	ND	ND	6.59 ± 1.10
	Quercetin-triglucoside	ND	ND	ND	0.92 ± 0.17	ND	ND	ND	0.58 ± 0.59	2.04 ± 0.12	ND	ND	ND
	Quercetin-triglucoside	ND	ND	ND	ND	ND	ND	ND	ND	2.36 ± 0.13	ND	ND	ND
	Quercetin-diglucoside	1.74 ± 0.66	1.00 ± 0.43	0.01 ± 0.05	0.03 ± 0.05	ND	ND	ND	0.05 ± 0.16	1.80 ± 0.62	ND	ND	0.09 ± 0.19
	Quercetin-diglucoside	ND	ND	ND	ND	ND	ND	ND	ND	36.17 ± 6.06	ND	ND	ND
	Quercetin-diglucoside	0.13 ± 0.02	0.32 ± 0.04	0.07 ± 0.07	6.00 ± 1.05	0.26 ± 0.16	0.07 ± 0.06	0.31 ± 0.44	0.91 ± 0.73	48.53 ± 9.68	ND	ND	1.42 ± 0.16
	Isohamnetin-3-*O*-rutinoside	ND	ND	ND	ND	ND	ND	ND	ND	ND	ND	ND	2.20 ± 0.52
	Isorhamnetin-glucoside	0.24 ± 0.05	0.06 ± 0.10	ND	ND	ND	ND	ND	ND	ND	ND	ND	ND
	Isorhamnetin-diglucoside	ND	ND	ND	ND	ND	ND	ND	ND	3.55 ± 0.52	ND	ND	ND
	Isorhamnetin-triglucoside	ND	ND	ND	ND	ND	ND	ND	ND	ND	ND	ND	0.88 ± 0.36
	Isorhamnetin-triglucoside	ND	ND	ND	ND	ND	ND	ND	ND	ND	ND	ND	1.39 ± 0.13
	Isorhamnetin-triglucoside	0.60 ± 0.18	0.10 ± 0.14	ND	ND	ND	ND	ND	ND	ND	ND	ND	0.27 ± 0.21
	Isorhamnetin-diglucoside	226.92 ± 57.15	112.07 ± 68.68	0.63 ± 0.61	ND	ND	ND	ND	0.32 ± 0.41	4.60 ± 2.18	ND	ND	0.58 ± 0.08
	Isorhamnetin-triglucoside	0.32 ± 0.12	0.27 ± 0.16	ND	ND	ND	ND	ND	ND	ND	ND	ND	ND
	Isorhamnetin-diglucoside	0.27 ± 0.08	0.09 ± 0.10	ND	ND	ND	ND	ND	0.88 ± 0.93	14.32 ± 5.88	ND	ND	ND
	Isorhamnetin-diglucoside	ND	ND	ND	ND	ND	ND	ND	ND	ND	ND	ND	119.05 ± 29.96
	Rutin	ND	ND	ND	ND	ND	ND	ND	ND	0.82 ± 0.94	ND	ND	126.57 ± 2.05
	Nicotiflorin (kaempferol-3-*O*-rutinoside)	ND	ND	ND	ND	ND	ND	ND	ND	ND	ND	ND	14.41 ± 3.33
	Quercetin	41.44 ± 14.56	92.23 ± 26.62	43.85 ± 12.40	53.83 ± 14.18	50.14 ± 18.80	50.98 ± 17.88	169.31 ± 36.21	128.76 ± 36.60	86.33 ± 27.25	65.83 ± 19.58	13.44 ± 7.36	87.24 ± 23.97
	Isorhamnetin	0.44 ± 0.19	1.61 ± 0.92	ND	ND	ND	ND	ND	ND	ND	ND	ND	ND
	Kaempferol-3-*O*-caffeoyl diglucoside-7-*O*-diglucoside	ND	ND	ND	ND	ND	ND	ND	ND	ND	ND	ND	0.66 ± 0.49
	Kaempferol-3-*O*-caffeoyl diglucoside-7-*O*-diglucoside	1.10 ± 0.42	0.15 ± 0.23	ND	ND	ND	ND	ND	ND	ND	ND	ND	ND
	Kaempferol-3-*O*-feruloyl triglucoside-7-*O*-glucoside	ND	ND	ND	7.26 ± 1.92	ND	ND	ND	0.10 ± 0.17	ND	ND	ND	ND
	Kaempferol-3-*O*-sinapoyl diglucoside-7-*O*-glucoside	26.25 ± 2.71	44.68 ± 7.12	0.18 ± 0.05	21.88 ± 5.56	1.51 ± 0.59	ND	ND	0.06 ± 0.19	ND	ND	ND	0.69 ± 0.14
	Kaempferol-3-*O*-feruloyl diglucoside-7-*O*-glucoside	31.88 ± 2.64	35.02 ± 5.37	ND	48.14 ± 11.44	ND	ND	ND	ND	ND	ND	ND	ND
	Kaempferol-3-*O*-*p*-coumaroyl diglucoside-7-*O*-glucoside	ND	ND	ND	ND	ND	ND	ND	ND	ND	ND	ND	1.13 ± 0.23
	Kaempferol-3-*O*-feruloyl triglucoside-7-*O*-glucoside	ND	0.02 ± 0.12	ND	ND	ND	ND	ND	ND	ND	ND	ND	ND
	Kaempferol-3-*O*-caffeoyl diglucoside-7-*O*-diglucoside	1.88 ± 0.70	ND	0.36 ± 0.37	44.00 ± 12.04	0.62 ± 0.46	ND	0.24 ± 0.45	5.10 ± 2.76	ND	ND	ND	ND
	Kaemperol-3-*O*-sinapoyl triglucoside-7-*O*-diglucoside	ND	ND	ND	0.19 ± 0.17	ND	ND	ND	ND	ND	ND	ND	ND
	Kaempferol-3-*O*-caffeoyl diglucoside-7-*O*-glucoside	598.05 ± 159.49	628.11 ± 178.38	0.34 ± 0.38	261.36 ± 66.57	1.66 ± 0.58	ND	3.15 ± 2.46	0.73 ± 0.45	3.35 ± 3.79	ND	ND	ND
	Kaempferol-3-*O*-caffeoyl diglucoside-7-*O*-glucoside	ND	ND	ND	8.72 ± 4.47	0.92 ± 1.04	ND	ND	3.07 ± 3.07	ND	ND	ND	ND
	Kaemperol-3-*O*-sinapoyl triglucoside-7-*O*-diglucoside	ND	ND	ND	22.07 ± 4.89	2.18 ± 1.64	ND	ND	0.92 ± 0.85	ND	ND	ND	ND
	Kaemperol-3-*O*-sinapoyl diglucoside-7-*O*-diglucoside	1.70 ± 0.55	ND	1.82 ± 1.38	52.63 ± 4.01	5.09 ± 2.18	4.44 ± 1.00	0.38 ± 0.48	5.28 ± 2.21	ND	ND	ND	ND
	Kaempferol-3-*O*-*p*-coumaroyl diglucoside-7-*O*-glucoside	96.58 ± 20.96	108.00 ± 20.50	ND	51.84 ± 18.65	ND	ND	0.44 ± 0.62	ND	0.05 ± 0.50	ND	ND	ND
	Kaempferol-3-*O*-caffeoyl diglucoside-7-*O*-diglucoside	1.78 ± 0.69	1.99 ± 0.93	ND	1.34 ± 0.36	ND	ND	ND	1.63 ± 1.29	ND	ND	ND	ND
	Kaempferol-3-*O*-caffeoyl diglucoside-7-*O*-diglucoside	ND	ND	ND	ND	ND	ND	ND	ND	ND	ND	ND	140.19 ± 30.20
	Kaempferol-3-*O*-feruloyl diglucoside-7-*O*-glucoside	ND	ND	ND	0.16 ± 0.22	ND	ND	ND	2.96 ± 1.70	ND	ND	ND	ND
	Kaempferol-3-*O*-feruloyl diglucoside-7-*O*-glucoside	ND	ND	ND	ND	ND	ND	ND	ND	ND	ND	ND	40.29 ± 24.09
	Kaempferol-3-*O*-sinapoyl diglucoside-7-*O*-glucoside	ND	ND	ND	ND	ND	ND	ND	ND	ND	ND	ND	54.08 ± 27.52
	Kaempferol-3-*O*-sinapoyl diglucoside	5.96 ± 1.28	10.64 ± 0.56	ND	10.78 ± 3.27	ND	ND	0.48 ± 0.70	0.07 ± 0.42	ND	ND	ND	6.09 ± 1.13
	Kaempferol-3-*O*-feruloyl diglucoside	0.16 ± 0.10	0.22 ± 0.17	ND	0.79 ± 0.65	ND	ND	ND	ND	ND	ND	ND	ND
	Quercetin-3-*O*-feruloyl diglucoside-7-*O*-diglucoside	ND	ND	ND	2.12 ± 0.68	ND	ND	ND	ND	ND	ND	ND	ND
	Quercetin-3-*O*-caffeoyl diglucoside-7-*O*-glucoside	18.15 ± 2.32	15.10 ± 6.93	ND	3.00 ± 0.75	0.09 ± 0.08	ND	0.04 ± 0.17	ND	ND	ND	ND	0.13 ± 0.02
	Quercetin-3-*O*-feruloyl diglucoside-7-*O*-glucoside	62.82 ± 18.68	91.35 ± 26.44	0.06 ± 0.09	29.24 ± 11.25	ND	0.04 ± 0.16	ND	ND	0.23 ± 0.38	ND	ND	ND
	Quercetin-3-*O*-feruloyl diglucoside-7-*O*-glucoside	9.90 ± 2.89	10.67 ± 4.75	ND	1.04 ± 0.31	ND	ND	ND	ND	ND	ND	ND	0.05 ± 0.02
	Quercetin-3-*O*-sinapoyl diglucoside	0.05 ± 0.03	0.28 ± 0.11	ND	1.07 ± 0.25	ND	ND	0.05 ± 0.20	ND	ND	ND	ND	19.91 ± 5.43
	Quercetin-3-*O*-sinapoyl diglucoside	ND	ND	ND	ND	ND	ND	ND	ND	0.55 ± 0.21	ND	ND	ND
	Quercetin-3-*O*-feruloyl diglucoside-7-*O*-diglucoside	0.07 ± 0.06	ND	ND	ND	ND	ND	ND	ND	ND	ND	ND	ND
Total flavonoids and derivatives		1400.25 ± 173.05	1415.71 ± 198.12	53.99 ± 12.54	780.53 ± 76.45	80.78 ± 19.61	63.01 ± 18.24	177.13 ± 36.34	167.30 ± 37.17	298.89 ± 33.09	65.83 ± 19.58	13.44 ± 7.36	739.20 ± 66.66
Total		5279.75 ± 673.23	8910.55 ± 992.69	11,157.73 ± 3638.45	25,275.21 ± 1819.35	40,270.79 ± 2754.00	47,835.56 ± 16,152.09	12,972.52 ± 2318.92	39,571.96 ± 7690.26	12,450.21 ± 3303.01	2282.31 ± 652.32	484.73 ± 244.95	10,449.67 ± 3350.06

**Table 2:** DPPH radical scavenging activity, oxygen radical absorbance capacity (ORAC) and total phenolic content (TPC) of cruciferous vegetables. Data are expressed as mean ± standard error of mean (SEM), (*n* = 3). Different letters (^a–f^) implies significant differences between groups in the same assay (*p* < 0.05).

Vegetable	Scientific Name	DPPH (μmol TE/g FW)	ORAC (μmol TE/g FW)	TPC (mg GAE/g FW)
Pakchoi	*B. rapa* var. *chinensis*	4.22 ± 0.41 ^c^	13.51 ± 2.35 ^bcd^	0.78 ± 0.16 ^cd^
Choysum	*B. rapa* var. *parachinensis*	3.84 ± 1.03 ^c^	11.97 ± 5.79 ^cd^	0.68 ± 0.20 ^cde^
Chinese cabbage	*B. rapa* var. *pekinensis*	1.32 ± 0.05 ^d^	3.45 ± 0.25 ^d^	0.21 ± 0.03 ^ef^
Kailan	*B. oleracea* var. *alboglabra*	6.83 ± 1.23 ^b^	23.73 ± 4.89 ^abc^	1.28 ± 0.19 ^b^
Brussels sprout	*B. oleracea* var. *gemmifera*	9.54 ± 0.77 ^a^	26.67 ±10.48 ^ab^	1.92 ± 0.24 ^a^
Cabbage	*B. oleracea* var. *capitata*	1.64 ± 0.24 ^d^	7.05 ± 1.55 ^d^	0.35 ± 0.03 ^def^
Cauliflower	*B. oleracea* var. *botrytis*	2.71 ± 0.75 ^cd^	9.53 ± 3.56 ^cd^	0.57 ± 0.06 ^cdef^
Broccoli	*B. oleracea* var. *italica*	3.85 ± 0.58 ^c^	23.09 ± 4.16 ^abc^	1.06 ± 0.12 ^bc^
Rocket salad	*E. sativa*	8.18 ± 1.20 ^ab^	32.08 ± 7.52 ^a^	1.93 ± 0.35 ^a^
Red cherry radish	*R. sativus*	2.70 ± 0.29 ^cd^	22.08 ± 3.47 ^abc^	0.68 ± 0.07 ^cde^
Daikon radish	*R. sativus*	1.11 ± 0.23 ^d^	5.34 ± 2.50 ^d^	0.16 ± 0.04 ^f^
Watercress	*N. officinale*	7.76 ± 0.46 ^ab^	32.92 ± 1.70 ^a^	1.44 ± 0.15 ^ab^

## Supplementary Materials

The Supplementary materials are available online.
