# Supplementary material for: Profiling of Phenolic Compounds and Antioxidant Activity of 12 Cruciferous Vegetables
Source: Molecules. 2018 May 10;23(5):1139. doi: 10.3390/molecules23051139 (PMC6100362; doi:10.3390/molecules23051139)
Supplement: Supplementary file 1 [file molecules-23-01139-s001.zip › molecules-297271-SI.pdf]

Supplementary data

Profiling of phenolic compounds and antioxidant activity of 12 cruciferous vegetables

Zhifeng Li[1†], Hui Wen Lee[2†], Xu Liang[2], Dong Liang[3], Qi Wang[1], Dejian Huang[4], Choon Nam Ong[2,3*]

[1] *National Pharmaceutical Engineering Center for Solid Preparation in Chinese Herbal Medicine, Jiangxi University of Traditional Chinese Medicine, No. 818 Yunwan Road, Nanchang 330002, China.*

[2] *NUS Environmental Research Institute, National University of Singapore, 5A Engineering Drive 1, Singapore 117411, Singapore.*

[3] *Saw Swee Hock School of Public Health, National University of Singapore, 12 Science Drive 2, Singapore 117549.*

[4] *Food Science and Technology Program, Department of Chemistry, National University of Singapore, 3 Science Drive 3, Singapore 117543, Singapore.*

[†]*These authors contributed equally to the work.*

Address correspondence to:

Choon Nam Ong, PhD

NUS Environment Research Institute,

National University of Singapore,

T-Lab Building, 5A Engineering Drive 1, Singapore 117411.

Tel: +65 6516-7386; Fax: +65-6779-1489; E-mail: eridir@nus.edu.sg

Table S1. Multiple reaction monitoring (MRM) conditions for various phenolic compound using UHPLC-QqQ-MS/MS.

**Retention time (min)**	**Peak**	**Peak identification**	**Class**	**[M−H]⁻ (*m/z*)**	**MS² Product ion (*m/z*)**	**Collision energy (V)**
6.7	1	5-Caffeoylquinic acid [a]	Hydroxycinnamic acids and derivatives	353	179	20
7.1	2	Quercetin-triglucoside [bd]	Flavonoids and derivatives	787	625	40
7.4	3	Quercetin-triglucoside [bd]	Flavonoids and derivatives	787	625	40
7.5	4	Quercetin-triglucoside [bd]	Flavonoids and derivatives	787	625	40
7.6	5	Kaempferol-3-O-feruloyltriglucoside-7-O-glucoside [bc]	Flavonoids and derivatives	1109	609	40
7.6	6	Kaempferol-glucoside [bc]	Flavonoids and derivatives	447	284	35
7.7	7	Kaempferol-triglucoside [bc]	Flavonoids and derivatives	771	609	20
7.9	8	Quercetin-3-O-caffeoyldiglucoside-7-O-glucoside [bd]	Flavonoids and derivatives	949	787	40
7.9	9	3-Caffeoylquinic acid [a]	Hydroxycinnamic acid derivative	353	191	20
8.0	10	Quercetin-3-O-feruloyldiglucoside-7-O-diglucoside [bd]	Flavonoid acylated with hydroxycinnamic acid	1125	771	55
8.0	11	4-Caffeoylquinic acid [a]	Hydroxycinnamic acids and derivatives	353	179	20
8.089	12	Kaempferol-3-O-caffeoyldiglucoside-7-O-diglucoside [bc]	Flavonoids and derivatives	1095	933	40
8.1	13	Kaempferol-3-O-sinapoyltriglucoside-7-O-diglucoside [bc]	Flavonoids and derivatives	1301	771	50
8.1	14	Quercetin-3-O-feruloyldiglucoside-7-O-glucoside [bd]	Flavonoids and derivatives	963	801	40
8.1	15	Quercetin-diglucoside [bd]	Flavonoids and derivatives	625	463	25
8.1	16	Caffeic acid [a]	Hydroxycinnamic acids and derivatives	179	135	10
8.1	17	3-Feruloylquinic acid [a]	Hydroxycinnamic acids and derivatives	367	173	10
8.168	18	Kaempferol-3-O-caffeoyldiglucoside-7-O-glucoside [bc]	Flavonoids and derivatives	933	609	45
8.3	19	Kaemperol-3-O-sinapoyltriglucoside-7-O-diglucoside [bc]	Flavonoids and derivatives	1301	977	50
8.3	20	Quercetin-3-O-feruloyldiglucoside-7-O-glucoside [bd]	Flavonoids and derivatives	963	801	40
8.4	21	Kaempferol-3-O-sinapoyldiglucoside-7-O-diglucoside [bc]	Flavonoids and derivatives	1139	977	40
8.5	22	Kaempferol-3-O-feruloyltriglucoside-7-O-glucoside [bc]	Flavonoids and derivatives	1109	947	40
8.5	23	Quercetin-diglucoside [bd]	Flavonoids and derivatives	625	463	25
8.521	24	Kaempferol-3-O-caffeoyldiglucoside-7-O-glucoside [bc]	Flavonoids and derivatives	933	609	45

8.6	25	Kaempferol-diglucoside [bc]	Flavonoids and derivatives	609	285	40
8.6	26	Kaempferol-3-O-sinapoyldiglucoside-7-O-glucoside [bc]	Flavonoids and derivatives	977	815	40
8.7	27	Kaempferol-3-O-feruloyldiglucoside-7-O-glucoside [bc]	Flavonoids and derivatives	947	785	40
8.7	28	Kaempferol-3-O-*p*-coumaroyldiglucoside-7-O-glucoside [bc]	Flavonoids and derivatives	917	755	38
8.7	29	Isorhamnetin-glucoside [be]	Flavonoids and derivatives	477	314	38
8.7	30	Isorhamnetin-diglucoside [be]	Flavonoids and derivatives	639	315	40
9.1	31	Quercetin-triglucoside [bd]	Flavonoids and derivatives	787	625	40
9.166	32	4-feruloylquinic acid (coeluted with 5-feruloylquinic acid) [a]	Hydroxycinnamic acids and derivatives	367	173	10
9.2	33	Quercetin-diglucoside [bd]	Flavonoids and derivatives	625	463	25
9.25	34	Kaempferol-diglucoside [bc]	Flavonoids and derivatives	609	285	40
9.4	35	Quercetin-triglucoside [bd]	Flavonoids and derivatives	787	625	40
9.4	36	Isorhamnetin-diglucoside [be]	Flavonoids and derivatives	639	315	40
9.4	37	*p*-Coumaric acid [a]	Hydroxycinnamic acids and derivatives	163	119	10
9.5	38	Kaempferol-3-O-caffeoyldiglucoside-7-O-diglucoside [bc]	Flavonoids and derivatives	933	609	45
9.67	39	Kaempferol-diglucoside [bc]	Flavonoids and derivatives	609	285	40
9.7	40	Kaempferol-3-O-caffeoyldiglucoside-7-O-diglucoside [bc]	Flavonoids and derivatives	933	609	
9.7	41	Quercetin-3-O-sinapoyldiglucoside [bd]	Flavonoids and derivatives	831	300	40
9.8	42	Iso-sinapic acid [bf]	Hydroxycinnamic acids and derivatives	223	149	10
9.9	43	Kaempferol-triglucoside [bc]	Flavonoids and derivatives	771	609	20
9.9	44	Quercetin-3-O-sinapoyldiglucoside [bd]	Flavonoids and derivatives	831	300	40
9.9	45	Isorhamnetin-triglucoside [be]	Flavonoids and derivatives	801	639	20
9.93	46	Rutin [a]	Flavonoids and derivatives	609	300	40
10	47	Ferulic acid [a]	Hydroxycinnamic acids and derivatives	193	134	10
10.1	48	Sinapic acid [a]	Hydroxycinnamic acids and derivatives	223	134	10
10.17	49	Kaempferol-3-O-sinapoyldiglucoside [bc]	Flavonoids and derivatives	815	609	40
10.18	50	Kaempferol-3-O-feruloyldiglucoside-7-O-glucoside [bc]	Flavonoids and derivatives	947	609	40
10.18	51	Quercetin-3-O-glucoside [a]	Flavonoids and derivatives	463	300	25
10.25	52	Kaempferol-3-O-caffeoyldiglucoside-7-O-diglucoside [bc]	Flavonoids and derivatives	1095	933	40
10.26	53	Kaempferol-3-O-sinapoyldiglucoside-7-O-glucoside [bc]	Flavonoids and derivatives	977	609	40

10.267	54	Quercetin-3-O-feruloyldiglucoside-7-O-diglucoside [bd]	Flavonoids and derivatives	1125	771	55
10.38	55	Kaempferol-3-O-feruloyldiglucoside-7-O-glucoside [bc]	Flavonoids and derivatives	947	609	40
10.4	56	Kaempferol-3-O-caffeoyldiglucoside-7-O-diglucoside [bc]	Flavonoids and derivatives	1095	933	40
10.4	57	kaempferol-3-O-p-coumaroyldiglucoside-7-O-glucoside [bc]	Flavonoids and derivatives	917	609	38
10.5	58	Nicotiflorin (kaempferol-3-O-rutinoside) [a]	Flavonoids and derivatives	593	255	20
10.6	59	Isorhamnetin-3-O-rutinoside [a]	Flavonoids and derivatives	623	315	40
10.7	60	Isorhamnetin-diglucoside [be]	Flavonoids and derivatives	639	315	40
10.8	61	Kaempferol-glucoside [bc]	Flavonoids and derivatives	447	284	35
10.8	62	Isorhamnetin-triglucoside [be]	Flavonoids and derivatives	801	639	20
10.9	63	Isorhamnetin-triglucoside [be]	Flavonoids and derivatives	801	639	20
11	64	Isorhamnetin-triglucoside [be]	Flavonoids and derivatives	801	639	20
11.2	65	1,2-Disinapoylgentiobiose [bf]	Hydroxycinnamic acids and derivatives	753	529	22
11.3	66	Isorhamnetin diglucoside [be]	Flavonoids and derivatives	639	315	40
11.4	67	Kaempferol-3-O-feruloyldiglucoside [bc]	Flavonoids and derivatives	785	609	40
11.4	68	1-Sinapoyl-2-feruloylgentiobiose [bf]	Hydroxycinnamic acids and derivatives	723	499	22
11.5	69	1,2-Diferuloylgentiobiose [bg]	Hydroxycinnamic acids and derivatives	693	499	20
12.1	70	1,2,2'-Trisinapoylgentiobiose [bf]	Hydroxycinnamic acids and derivatives	959	205	40
12.3	71	1,2'-Disinapoyl-2-feruloylgentiobiose [bf]	Hydroxycinnamic acids and derivatives	929	705	35
12.5	72	1-Sinapoyl-2-2'diferuloylgentiobiose [bg]	Hydroxycinnamic acids and derivatives	899	705	40
12.7	73	Quercetin [a]	Flavonoids and derivatives	301	151	20
14.3	74	Isorhamnetin [a]	Flavonoids and derivatives	315	300	20

[a] Peak was identified by matching the retention time and *m/z* value with authentic standard.

[b] Tentative identification was obtained by comparing their elution order, molecular ions $[M-H]^-$ and MS^2 fragments with literature data (Lin & Harnly, 2009, 2010)

[c] Kaempferol glycosides were semi-quantified using cynaroside (luteolin-7-O-glucoside)..

[d] Quercetin glycosides were semi-quantified using isoquercitrin (quercetin-3-O-glucoside).

[e] Isorhamnetin glycosides were semi-quantified using narcissin (isorhamnetin-3-O-rutinoside).

[f] Sinapic acid derivatives were semi-quantified using sinapic acid.

[g] Ferulic acid derivatives were semi-quantified using ferulic acid.

Table S2. Method validation for the quantification of phenolic compounds using UHPLC-QqQ-MS/MS.

Phenolic compounds	Calibration curve		Linear range (ng/ml)	LOD	Reproducibility	Recovery (%)	
	Regression equation	R^2		(ng/ml)	(RSD, %)	Low	High
p-coumaric acid	y = 36.229x + 0.495	0.9977	1−1000	0.05	1.74	91.14	109.41
Ferulic acid	y = 3.6407x - 0.004	0.9997	1−1000	0.21	5.36	74.64	112.15
Sinapic acid	y = 1.3546x + 0.0127	0.9984	1−1000	0.09	5.95	92.15	94.75
Caffeic acid	y = 433.15x + 2.3382	0.9988	1−1000	0.45	3.53	75.21	108.43
3-caffeoylquinic acid	y = 464.7x + 0.0084	0.9998	1−1000	0.07	3.38	72.34	97.41
4-caffeoylquinic acid	y = 142.61x - 0.2893	0.9999	1−1000	0.03	2.78	111.31	91.53
5-caffeoylquinic acid	y = 302.38x - 1.2588	0.9990	1−1000	0.02	1.56	81.61	97.67
3-feruloylquinic acid	y = 14.067x - 0.0479	0.9991	1−1000	0.15	0.89	91.22	113.13
4-feruloylquinic acid	y = 536.81x - 0.3827	0.9996	1−1000	0.01	1.96	71.63	97.48
Quercetin	y = 33.304x + 0.71	0.9963	1−1000	0.24	1.68	82.34	110.38
Rutin	y = 200.53x + 0.8473	0.9988	1−1000	0.11	4.88	72.42	104.08
Narcissin	y = 175.7x + 0.713	0.9997	1−1000	0.02	6.24	110.13	96.37
Kaempferol-3-O-rutinoside	y = 0.1874x + 0.0013	0.9994	1−1000	0.03	4.25	101.72	92.59
Kaempferol	y = 0.7569x + 0.0088	0.9982	1−1000	0.01	5.83	94.73	109.06
Quercetin-3-O-rhamnoside	y = 158.79x + 0.1105	0.9984	1−1000	0.06	6.48	92.02	103.32
Quercetin-3-O-glucoside	y = 452.11x + 2.0232	0.9987	1−1000	0.04	6.38	74.29	108.98

Isorhamnetin	y = 141.72x + 1.0964	0.9984	1−1000	0.02	2.02	73.28	102.39
